# Effect of Electrospun Non-Woven Mats of Dibutyryl Chitin/Poly(Lactic Acid) Blends on Wound Healing in Hairless Mice

**DOI:** 10.3390/molecules17032992

**Published:** 2012-03-09

**Authors:** Seon Il Jang, Ji Ye Mok, In Hwa Jeon, Kwang-Hyun Park, Thuy Thi Thu Nguyen, Jun Seo Park, Hee Min Hwang, Mi-Sun Song, Duckhee Lee, Kyu Yun Chai

**Affiliations:** 1 School of Alternative Medicine and Health Science, College of Alternative Medicine, Jeonju University, Jeonju 560-759, Korea; Email: sonjjang@jj.ac.kr (S.I.J.); treehouse@hanmail.net (J.Y.M.); inflowerj@hanmail.net (I.H.J.); 2 Department of Biochemistry, Chonbuk National University Medical School, Jeonju 561-756, Korea; Email: khpark@chonbuk.ac.kr; 3 Center of Chemical Technology, Division of Chemical Engineering, Hankyong National University, 167 Chungang-ro, Anseong-si, Gyeonggi-do 456-749, Korea; Email: nt.thui82@gmail.com (T.T.T.N.); jspark@hknu.ac.kr (J.S.P.); 4 Division of Bio Nano Chemistry, College of Natural Sciences, Wonkwang University, Iksan 570-749, Korea; Email: proteen00@naver.com (H.M.H.); misun4y@nate.com (M.-S.S.); dl202@wonkwang.ac.kr (D.L.)

**Keywords:** dibutyryl chitin, nanofiber membrane, electrospinning, hairless mice, wound healing

## Abstract

The aim of this study was to examine the proliferative ability of dibutyryl chitin (DBC) on scratch wounds in HaCaT keratinocytes and to evaluate the effect of nanoporous non-woven mat (DBCNFM) on skin wound healing in hairless mice using the advantages of DBCNFM, such as high porosity and high surface area to volume. The cell spreading activity of DBC was verified through a cell spreading assay in scratched human HaCaT keratinocytes. Scratch wound experiments showed that DBC notably accelerates the spreading rate of HaCaT keratinocytes in a dose dependent manner. The molecular aspects of the healing process were also investigated by hematoxylin & eosin staining of the healed skin, displaying the degrees of reepithelialization and immunostaining on extracellular matrix synthesis and remodeling of the skin. Topical application of DBCNFM significantly reduced skin wound rank scores and increased the skin remodeling of the wounded hairless mice in a dose dependent way. Furthermore, DBCNFM notably increased the expression of the type 1 collagen and filaggrin. These results demonstrate that DBC efficiently accelerates the proliferation of HaCaT keratinocytes and DBCNFM notably increases extracellular matrix synthesis on remodeling of the skin, and these materials are a good candidate for further evaluation as an effective wound healing agent.

## 1. Introduction

Skin is the largest organ of the integumentary system in the body and normally separates and protects the internal structures from the external environment. The structure of skin is composed of three layers such as epidermis, dermis and hypodermis. The outer skin, the epidermis, is a tightly bound, stratified, and squamous epithelium, covered by keratin [1,2,3]. Barrier function of human skin largely depends on the final product of epidermal cell differentiation, the horny layer, *i.e.*, stratum corneum [2,3]. The middle layer is called the dermis, which is composed of connective tissue, blood vessels, nerve endings, hair follicles, and sweat and oil glands.

Electrospinning is known as an efficient tool to fabricate multifunctional fibrous matrices, which are composed of polymeric fibers with diameters ranging from hundreds of nanometers to several micrometers [4]. Nanofibrous matrices have applications such as tissue engineering, drug delivery, and wound dressing due to their large surface area to volume ratio, high porosity, and small pore size [5]. These electrospun porous matrices can mimic the micromorphology of the native extracellular matrix (ECM). For use in biomedical applications including wound dressing, the electrospun nanoporous matrices have to contain the chemical composition of the native ECM [6]. ECM molecules, which fill the inter-keratinocyte spaces in living epidermal layers, are involved in tissue hydration, nutrition, and regulation of cell proliferation and differentiation [7]. ECM molecules often combine in order to attain a higher degree of spatial organization leading to the formation of basement membranes [8]. If the skin is injured, irrespective of the size and severity of the injury, it should repair rapidly and perfectly. The healing response by the body’s self-treatment system starts right at the moment an injury occurs [3]. Filaggrin (FLG) associates with keratin intermediate filaments and facilitates the formation of the intracellular fibrous matrix during the transition from keratinocytes to corneocytes, which constitutes the cornified layer of the epidermis. Filaggrin is extensively deaminated at multiple residues in the early corneocyte [9]. Because of the lower affinity of deaminated filaggrin for keratins, it was proposed that filaggrin deamination facilitates its dissociation from the matrix and subsequent proteolysis [9,10].

Wound healing is a complex biological process coordinating a variety of cellular activities such as inflammation, migration to the wounded area, proliferation, angiogenesis and deposition and remodeling of extracellular matrix, mainly the collagen lattice [11]. A plethora of materials for wound dressing, skin substitutes, and recombinant growth factors have been shown to enhance the healing process, and some of them have been introduced into the clinical setting with therapeutic efficacy [12]. Many natural products are also known to possess wound healing properties, based on both anecdotal and scientific evidence [13,14]. Hence, there is a great interest in finding new wound healing products.

Among the different biodegradable polymers, poly(lactic acid) (PLA) not only has high mechanical strength, but also has biocompatibility and good cell attachment and proliferation. PLA has also good electrospinnability [15]. Chitin is the most widespread aminopolysaccharide in Nature and a major structural constituent of the exoskeletons of crustaceans and insects. Although chitin has been applied in pharmaceutics due to its specific physiochemical and biological properties [16], the low solubility of chitin has strongly restricted its technological applications. Recently, dibutyrylchitin (DBC) has been identified as a technologically friendly polymer with good solubility in several organic solvents [17,18,19,20,21,22,23,24,25]. DBC, a derivative of chitin, has useful biological properties such as antimicrobial activity and wound healing, but it also has lower mechanical properties and poor electrospinnability. To overcome of these shortcomings, electrospinning of a blended solution of PLA and DBC was conducted.

In this study, we fabricated a mechanically strong nanoporous non-woven mat of PLA/DBC nanofibers by an electrospinning process. We examined the proliferative ability off the non-woven mat of DBC on scratch wounds in HaCaT keratinocyte s and the effect of DBCNFM on skin wound healing in hairless mice. We found that DBC notably accelerates the spreading rate and collagen type 1 synthesis of HaCaT keratinocytes and topical application of DBCNFM significantly increases the skin remodeling in hairless mice. These results indicate that the DBCNFM can be used as a dressing material for wound healing.

## 2. Results

### 2.1. Morphology of Electrospun DBCNFM Composed of DBC/PLA Blend Nanofibers

**Figure 1:**
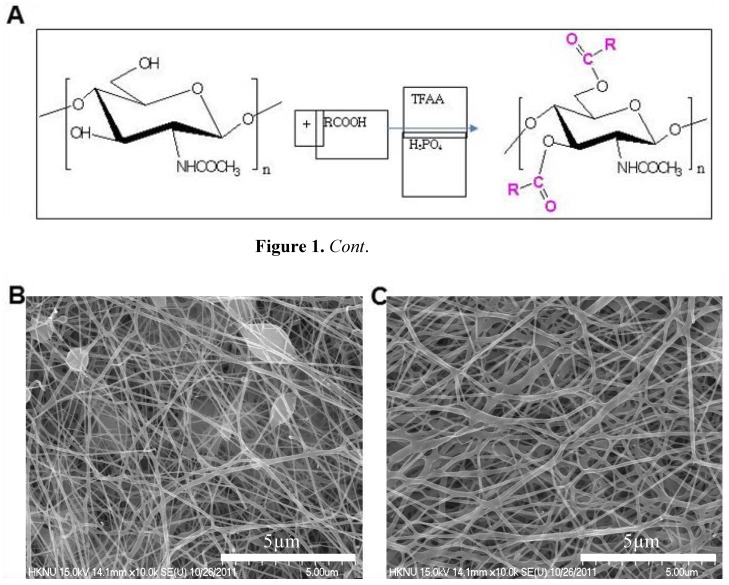
Structure of dibutyryl chitin (DBC) and FE-SEM pictures of DBCNFM—Nano-porous non-woven mats of electrospun DBC/PLA blend nanofibers. (**A**) DBC; R = R: CH_3_-CH_2_-CH_2_; (**B**) DBC/PLA (60/40) blend nanofibers and (**C**) DBC/PLA (30/70) blend nanofibers.

Figure 1B shows SEM pictures of porous non-woven mat of DBC/PLA nanofibers. The non-woven mat of DBC/PLA (60/40) blend nanofibers, shown in Figure 1C, had nanofibers with beads, while the non-woven mat of DBC/PLA (30/70) had smooth nanofibers. Higher content of DBC in the blend nanofibers prevents the formation of smooth nanofibers due to the cationic properties of DBC.

**Figure 2:**
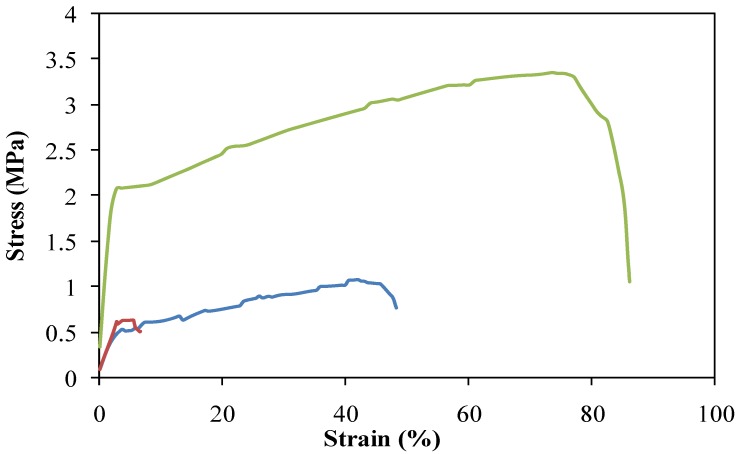
Tensile strength curves of DBCNFM—Nano-porous non-woven mats of electrospun. (Green line) PLA nanofibers, (Red line) DBC/PLA (60/40) blend nanofibers and (Blue line) DBC/PLA (30/70) blend nanofibers.

Figure 2 shows the tensile strength with elongation of the non-woven mats. PLA is known as a mechanically strong polymer. With the inclusion of DBC in the blends, the mechanical strength of non-woven mats was dramatically decreased. Non-woven mat of DBC/PLA (30/70) blend nanofibers had enough mechanical strength for experiments of the wound dressing, compared with that of DBC/PLA (60/40).

### 2.2. Effects of DBC on Spreading Rate of HaCaT Keratinocytes

**Figure 3:**
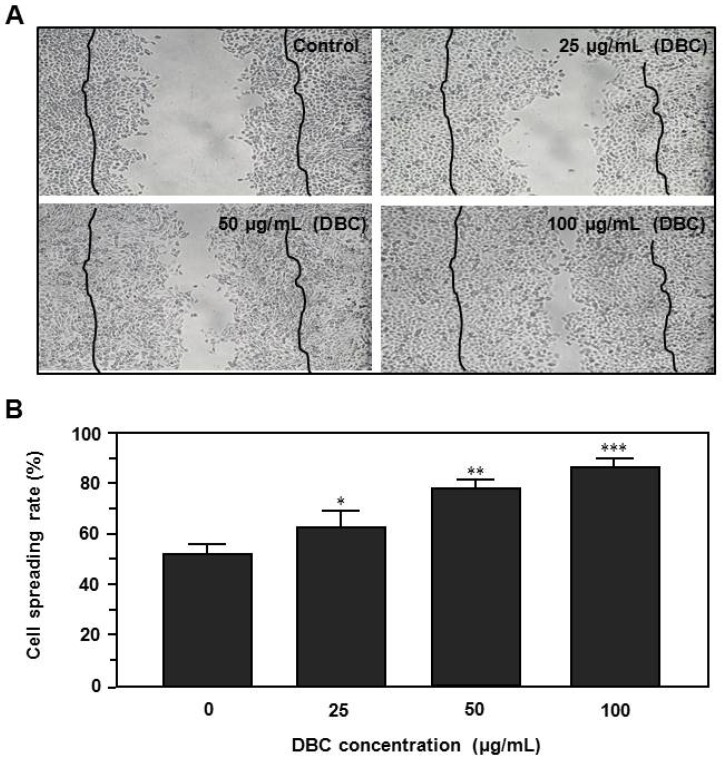
Effects of the DBC on the scratch wound healing of human HaCaT confluent monolayers. Cells cultured in 50 mm culture dish were mechanically scratched with a sterile 200 μL pipet tip and the wells were washed twice with PBS, and the cells were incubated with or without DBC (25–100 μg/mL), in triplicate for 24 h. Cell spreading morphology (**A**) and rate (**B**) were analyzed using the photographs from the scratched cell monolayers. Lines were the original scratching range. Magnification: 10×. The data are presented as the means ± S.D. of three independent experiments. * *p* < 0.05, ** *p* < 0.01 and ** *p* < 0.001 versus untreated control group.

First, we investigated the effects of DBC on spreading rate in human HaCaT keratinocytes. Cells were plated at 1 × 10^4^ per mL in 50-mm culture dish and cultured until the cells reached 70% confluence. HaCaT monolayers were mechanically scratched and exposed to DBC (25–100 µg/mL) for 24 h. As shown in Figure 3A, untreated control cells were growth (about 90% confluence) and spread (about 50%).

In contrast, cells exposed to DBC showed significantly higher spreading rates compare to controls in a dose dependent manner (Figure 3B). Especially, cells were markedly promoted the spreading by 100 µg/mL DBC for 24 h (*p* < 0.001)*.*

### 2.3. Effects of DBC on Collagen Synthesis of HaCaT Keratinocytes

**Figure 4:**
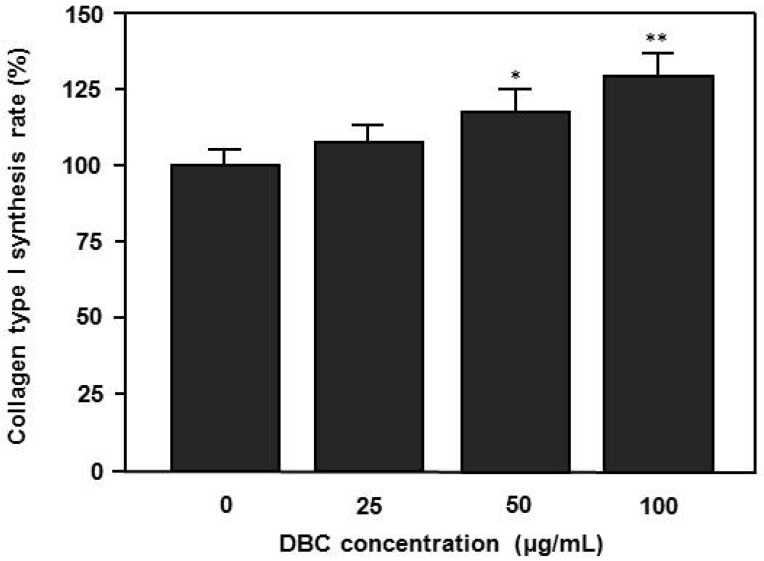
Effects of the DBC on collagen production in human HaCaT keratinocytes as determined by ELISA. Cells were seeded at 1× 10^4^ per well in 96 well plates and cultured until the cells reached 70% confluence and then exposed to various concentrations of DCE for 24 h. Collagen type 1 level was analyzed with ELISA after 24 h. The data are presented as the means ± S.D. of three independent experiments. * *p* < 0.05 and ** *p* < 0.01 versus untreated control group.

Next, we investigated the effects of DBC on collagen type I synthesis in human HaCaT keratinocytes. Cells were seeded at 1 × 10^4^ per well in 96 well plates and cultured until the cells reached 70% confluence and then exposed to various concentrations (25–100 µg/mL) of DBC for 24 h. As shown in Figure 4, DBC has the ability of inducing the synthesis of collagen type I of human HaCaT keratinocytes in a dose dependent manner. Also in this case, the maximum effect was found at a concentration of 100 µg/mL DCE (*p* < 0.01).

### 2.4. Effects of DBCNFM on Wound Healing Rank in Hairless Mice

**Figure 5:**
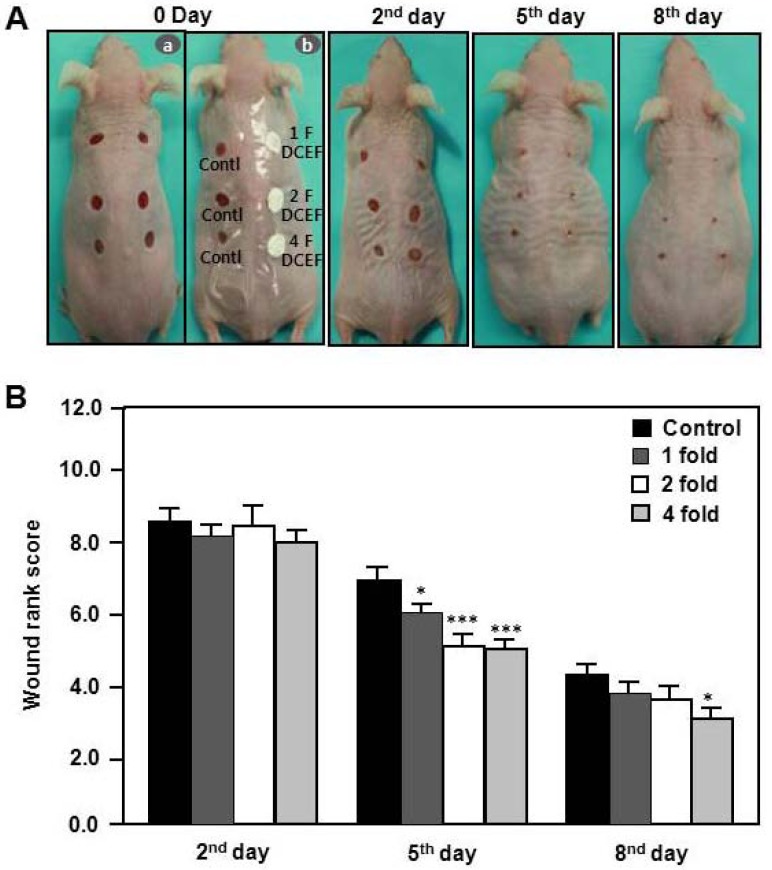
Effects of DBCNFM on wound healing in hairless mice. Wounds with 4 mm pore size were generated by punctures on the top, middle, and lower the back of hairless mice under anesthetization. Wounds were treated with or without DBCNFM and then sutured with Tegaderm. The macroscopic differences in healing were quantified using a wound rank scoring system, which assessed a wound closure by measuring wound length and gape, inflammation, redness, and swelling. (**A**) Macroscopic appearance of wounds (**B**) wound rank score on untreated control, DBCNFM treated groups (1–4 fold) on the 0, 2nd, 5th and 8th day. The data are presented as the means ± S.D. of three independent experiments; * *p* < 0.05 and *** *p* < 0.01 *versus* untreated control group.

To find effect of DBCNFM on the wound healing, we investigated the changes in the macroscopic appearance in hairless mice. Wound appearance, including the size variation of the cutaneous open wounds were daily monitored for wounds treated with or without DBCNFM (5 mm diameter, 1–4 fold) and then sutured with Tegaderm. Changes in macroscopic appearance were checked by capturing digital images of each animal at each time point of the 2nd, 5th or 8th day post wounding (Figure 5A).

Until the 5th day, the DBCNFM treatment dose dependently improved the healing rates and the macroscopic appearance of the cutaneous open wounds. The wounds in the topical DBCNFM-treated groups appeared to be less moist and inflamed and exhibited reduced wound areas, as compared with those in the non-treated control group. On the 8th day, the control rats demonstrated little contraction of wound surface and significant inflammation.

As compared with the wounds of control mice, the topical DBCNFM-treated wounds were smaller and more contracted. The topical DBCNFM-treated groups did not show skin blistering, which signifies the separation of the outer layer (epidermis) of the skin from the fiber layer (dermis). However, the wounds of the control mice were still in the process of wound contraction and were still inflamed. With regard to the macroscopic appearance in wound healing, the treatment efficiency of the 2-fold and 4-fold DBCNFM-treated mice on the 5th day appeared to be similar to that of the control mice on the 8th day.

The macroscopic appearance of wounds in the control and DBCNFM-treatment groups was blindly assessed via a wound rank scoring system that scored the degree of wound closure (wound length and gape) and inflammation (redness and swelling). On the 2nd day, the wound rank scores were not different between non-treated control group and DBCNFM-treated group. However, the wound rank scores on the 5th day were significantly reduced in the DBCNFM-treated groups compared to the non-treated control group in a dose dependent way. As shown in Figure 5B, the statistical analysis indicated a marked improvement of wound healing by the 5th day (*p* < 0.05 and *p* < 0.001).

### 2.5. Effects of DBCNFM on Histological Changes and ECM Expression in the Wounded Skin Tissues

**Figure 6:**
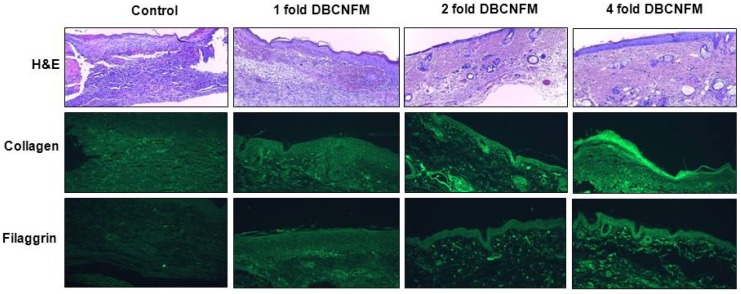
Representative micrographs of wound healing in the DBCNFM topical treated hairless mouse skin. Wound generation was punctured with 4 mm pore size on ahead, middle, and lower the back of hairless mice under anesthetization. Wounds were treated with or without DBCNFM (1–4 fold) and then sutured with tegaderm. On 5th day, the skin tissues were cut from wounded area and then used in H&E and immunohistochemical staining. Magnification: 100×.

To histologically evaluate the inhibitory effect of DBCNFM on wound healing, we performed H&E and immunohistochemical staining on specimens of the skins in the wounded hairless mice. On the 5th day, sections of the skin in the non-treated control group showed a large number of infiltrated inflammatory immune cells, including mononuclear cells and eosinophils (Figure 6).

In contrast, sections of the skins in the topical application of DBCNFM showed markedly diminished cellular infiltration into the dermis and recovered epidermal layer in a dose dependent fashon. We also conducted immunohistochemistry for ECM (type 1 collagen and filaggrin) to assess whether the topical application of DBCNFM has an effect on type 1 collagen and filaggrin expression in the wounded skin tissues. As shown in Figure 6, type 1 collagen and filaggrin were slightly expressed in the skin tissues of the non-treated control group. In contrast, they were markedly increased with the the topical application of DBCNFM in dose dependent manner.

**Figure 7:**
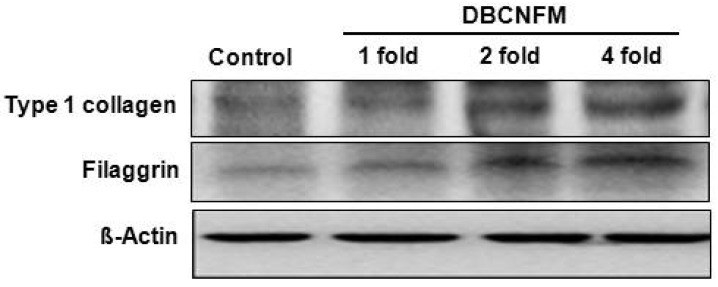
Effect of the DBCNFM on type 1 collagen and filaggrin expression in hairless mouse skin. Wound generation was accomplished with 4 mm pore size punctures on the top, middle, and lower the back of hairless mice under anesthetization. Wounds were treated with or without DBCNFM (1–4 fold) and then sutured with Tegaderm. On 5th day, the skin tissues were cut from wounded area and then used in Western bolt assays.

Finally, to evaluate the ECM expression by the topical application of DBCNFM on wound healing, we performed Western blot assays in the skin tissue lysates using anti- type 1 collagen and filaggrin antibodies. In parallel with immunohistochemical results, treatment with DBCNFM increased expression of type 1 collagen and filaggrin in the wounded skin tissues (Figure 7). These results demonstrate that the topical application of DBCNFM notably increases extracellular matrix synthesis on remodeling of the skin.

## 3. Discussion

Chitin accelerates the repair of different tissues, facilitates the contraction of wounds and regulates secretion of inflammatory mediators such as IL-1, IL-8 and PGE_2_ [26]. Electrospinning has been widely studied because of its efficiency and simplicity in fabricating nanofibrous structures [27]. A nano-porous non-woven mat, DBCNFM, exhibited outstanding characteristics, such as very large surface area-to-volume ratio and high porosity with a very small pore size. Therefore, the DBCNFM might be a good candidate for many biomedical applications, such as wound dressing, drug delivery and scaffolds for tissue engineering [28,29]. Recently, we synthesized DBC and fabricated DBCNFM by electrospinning a mixture of PLA and DBC, which could be applicable for biological applications. When DBCNFM was fabricated by electrospinning, 40/60 DBC/PLA blends it was found to give good mechanical strength and smooth nanofibers. The non-woven mat, made of these blend nanofibers was employed for the characterization of biological properties.

In this study, we demonstrate that DBC notably accelerated spreading rate and type 1 collagen production of HaCaT keratinocytes. We also found that topical application of DBCNFM significantly increased the skin remodeling in hairless mice through induction of type 1 collagen and filaggrin expression. Wound healing is a complex biological process, including inflammation, cell migration, angiogenesis, extracellular matrix synthesis, collagen deposition, re-epithelialization, *etc.* In the first half phase of the wound healing process, there are two major stages. The first is the inflammatory stage and the second is new tissue generation. At the inflammatory stage, infiltrating neutrophils cleanse foreign agents in the area, accelerating wound cleaning [30]. In previously studies, we demonstrated that DBC significantly inhibited the overproduction of the inflammatory mediators such as nitric oxide, prostaglandin E_2_ and pro-inflammatory cytokines (submission). During new tissue formation, the local accumulation of collagen strongly correlates with the accretion of tensile strength; hence, measuring the content and concentration of collagen at a repair site permits an estimate of the healing rate [31]. In this study, cells exposed to DBC showed significantly higher spreading rates and collagen synthesis compared to controls in a dose dependent manner (Figure 3 and Figure 4). These results suggest that DBC could be a potential candidate for spreading of human HaCaT keratinocytes, which would be particularly useful for wound healing and regeneration of skin.

It is known that chitin derivatives with quaternary ammonium groups possess high efficacy against bacteria and fungi. It is now widely accepted that the target site of these cationic polymers is the cytoplasmic membrane of bacterial cells [32]. Micro- and nanofibrous materials are suitable for preparing wound dressings. The best biomaterials for wound dressing should be biocompatible and promote the growth of dermis and epidermis layers. Chen *et al*. [33] reported composite nanofibrous membranes of chitosan/collagen, which are known for their beneficial effects on wound healing. The membranes were found to promote wound healing and induce cell migration and proliferation. Collagen is a major natural extracellular matrix component, and possesses a fibrous structure with fiber bundles varying in diameter from 50 to 500 nm [34]. Many efforts have been made to find an alternative scaffold material with similar physicochemical and biological characteristics as ECM [35]. The morphology of electrospun nanofiber mats is very similar to the morphology of human native ECM [36]. Filaggrin is a crucial component of the cornified cell envelope in the outer layer of the epidermis [37]. These skin barrier defects increase the risk of early onset of severe and persistent forms of wound-related skin diseases. To find the effects of DBCNFM on wound healing, we investigated the changes of macroscopic appearance in hairless mice. With regard to the macroscopic appearance in wound healing, the treatment efficiency of the 2-fold and 4-fold DBCNFM-treated mice on the 5th day appeared to be similar to that of the control mice on the 8th day (Figure 5).

On the 5th day, sections of the skin in the non-treated control group infiltrated a large number of infiltrated inflammatory immune cells including mononuclear cells and eosinophils but sections of the skins in the topical application of DBCNFM showed markedly diminished cellular infiltration into the dermis and recovered epidermal layer in a dose dependent way (Figure 6). The expression of collagen type 1 and filaggrin were markedly increased with the the topical application of DBCNFM in a dose dependent manner. Furthermore, in parallel with immunohistochemical results, treatment with DBCNFM increased expression of type 1 collagen and filaggrin in the wounded skin tissues (Figure 6 and Figure 7). These results demonstrate that the topical application of DBCNFM notably increases extracellular matrix synthesis on remodeling of the skin. However, the wound healing mechanism of DBCNFM is still obscure. Future investigations will thus be focused on the growth factor signaling pathways.

## 4. Experimental

### 4.1. Materials and Methods

^1^H-NMR spectra of the products were recorded at room temperature (20 °C) on a JEOL JNM-ECP 500 NMR spectrometer (500 MHz) using 5 mm diameter tubes. Samples were dissolved in DMSO-*d6* at the concenturation of 15 mg/ml. FT-IR spectra were recorded on a Shimadzu Prestige-21 FT-IR spectrometer, as described previously [38].

#### 4.1.1. Reagents

Dulbeco’s Modified Eagle’s Medium (DMEM), fetal bovine serum (FBS), and antibiotics were purchased from Gibco BRL (Grand Island, NY, USA). Antibodies against collagen type I and filaggrin were obtained from were obtained from R&D Systems (Minneapolis, MN, USA). Heamatoxylin & eosin, 3-(4,5-dimethylthiazol-2-yl)-2,5-diphenyltetrazolium bromide, a yellow tetrazole, MTT), averin (2,2,2-tribromoethanol) and other reagents were purchased from Sigma (St. Louis, MO, USA).

#### 4.1.2. Identification of DBC

The synthesized DBC was identified by IR and NMR spectroscopy as follows [39]. IR (KBr pellet, cm^−1^); N–H (3,100), C–H (3000–2890), C=O (1740), O=CNH (1685 and 1590), –CH_2_ (1395), –CH_3_ (1340), C–O (1200 and 1080). ^1^H-NMR (DMSO-d_6_, δ ppm); CON*H* (7.92, br), C_1_*H*-C_6_*H* (3.30–5.20, m), O (CO)C*H*_2_ at the C3 and C6 ester unit, respectively (2.14 and 2.30, br), NH(CO)C*H*_3_ (1.70, s, br), O(CO)CH_2_C*H*_2_ at the C3 and C6 ester unit, respectively (1.60 and 1.48, s), O(CO)CH_2_CH_2_C*H*_3_ at the C3 and C6 ester unit, respectively (0.90 and 0.80, 2 peaks, br) (Figure 1A).

#### 4.1.3. Fabrication of DBCNFM

12 wt% PLA solution in TFA was blended with 4 wt% DBC solution (15 wt% acetic acid/TFA). The weight ratios of polymer solutions in the DBC/PLA blend were 60/40 and 30/70, respectively. Electrospinning was carried out using blended polymer solutions. Each of the prepared solutions was poured into a standard 5-mL plastic syringe that was attached to a blunt 22-gauge stainless steel hypodermic needle. The solution flow rate was controlled using a syringe pump. A piece of Al sheet was wrapped around a rotating collector that was connected to the negative electrode. A high supply voltage (Chungpa EMI, Kunpo, Korea) was applied to the hypodermic needle as a positive electrode. The polymer solution was electrospun at a positive voltage of 16 kV, needle tip-to-collector distance of 12 cm, and solution flow rate of 5 µL/min.

#### 4.1.4. Characterization of DBCNFM

The morphology of the non-woven mats was investigated by FE-SEM (HITACHI S-4700, Tokushima, Japan) equipped with a BAL-TEC MED020 coating system. The average diameter of the electrospun fiber, its distribution, and its standard deviation were determined from measurements performed for about 110 fibers on the FE-SEM photographs by using visualization software (TOMORO Scope Eye 3.6). The mechanical property of the DBCNFM was characterized by tensile tester (LR 5K, LLOYD Instrument). The non-woven webs were tested with a 0.1 N preload at a cross-head speed of 5 mm/min. The length, width, and depth of test sheets of the non-woven webs were about 30 mm, 10 mm and 50 µm, respectively. The average values from five repetitions were taken as the tensile strength and elongation at break results.

### 4.2. Cell Line and Culture

Human HaCaT keratinocytes were obtained from the American Type Culture Collection (Rockville, MD, USA). HaCaT cells were cultured with DMEM medium containing 10% heat-inactivated FBS, penicillin G (100 IU/mL), and streptomycin (100 µg/mL) and incubated at 37 °C in a humidified atmosphere containing 5% CO_2_ and 95% air.

### 4.3. Scratch and Cell Spreading Assay

HaCaT cells harvested by trypsinization were seeded in 50 mm culture plates at a concentration of 1 × 10^4^/mL and cultured until the cells reached 70% confluence. A scratch was gently introduced in the center of the cell monolayers using a sterile 200 µL pipette tip. To remove the cell debris, the wells were washed twice with PBS, and the cells were incubated with or without DBC (25–100 µg/mL), in triplicate for 24 h. Cell spreading was analyzed using the photographs from the scratched cell monolayers. To ensure a representative count, each sample was divided into quarters, and two fields per quarter were photographed with an Olympus BX51 microscope at 10×. Cells that adopted a flattened, polygonal shape with filopodia- and lamellipodia-like extensions were considered to be spreading cells. In contrast, cells that resisted washing and remained tethered to the plate surface were considered to be non-spreading cells. The percentage of cells displaying a spreading morphology was quantified by dividing the number of spread cells by the total number of bound cells.

### 4.4. Collagen Type I Assay by ELISA

Type 1 collagen production was evaluated by enzyme-linked immunosorbent assay (ELISA). Briefly, cells were seeded at 1 × 10^4^ per well in 96 well plates and cultured until the cells reached 70% confluence and then exposed to various concentrations of DBC for 24 h. The medium was then removed and cells were washed once with PBS, fixed for 10 min with 4% formaldehyde, washed thrice with wash buffer (PBS, 0.5 mM CaCl_2_, 1 mM MgCl_2_, 0.1% Triton) followed by a blocking step of 30 min with 3% BSA in wash buffer. The mouse monoclonal antibody against cow type 1 collagen (Abcam, Cambridge, UK), diluted 1:300 in wash buffer containing 1% BSA, were added to wells and incubated for 2 h at room temperature under mild agitation. After this step, and after each of the following ones, the plates were emptied and repeatedly washed with wash buffer, to remove the excess of reagents. The horseradish peroxidase-labelled secondary antibody (Sigma, St. Louis, MO, USA), diluted 1:1,000 in PBS, were added to wells for 1 h at room temperature under mild agitation. The freshly prepared peroxidase substrate system for ELISA (3,3′,5,5′-tetramethylbenzidine, TMB) were added to microplates, and after 5min incubation the reaction products were read at 620 nm in the microplate reader.

### 4.5. Animals

Male hairless 6-week-old mice were purchased from Central Lab. Inc. (Seoul, Korea), the Korean branch of Charles River Japan (Kanagawa, Japan). The mice were maintained in an environmentally controlled rearing system and used for experiments when 8-weeks old. All experiments in this study were performed in accordance with Jeonju University Institutional Animal Care and Use Committee guidelines.

### 4.6. Induction of Wound

To find effect of DBCNFM on the wound healing, we used hairless mice. Four mm pore size (diameter) wounds were punctured on the top, middle, and lower back of hairless mice using a puncher (Natsume, Tokyo, Japan) under anesthetization by avertin. After the wound generation, the incisions were dressed with 70% ethanol. Wounds were treated with or without DBCNFM (5 mm diameter, 1–4 fold) and then sutured with Tegaderm (3M Health Care, Neuss, Germany). After topical application of DBCNFM, the mice were allowed to recover from anesthesia and each mouse was then returned to its cage.

### 4.7. Wound Rank Scoring System

The macroscopic differences in healing were quantified using a wound rank scoring system [40], which assessed a wound closure by measuring wound length and gape, inflammation, redness, and swelling. Measurements of wound gape and length at each time point were divided into four categories according to the best and worst reading, and on this basis, the wound was given a score of 0–3 for each measurement. Redness and swelling were also individually scored on a scale of 0–3 (no, slight, significant, or extensive redness/swelling). The four scores were then summed. The minimum score of 0 indicates the best possible condition of a wound, and the maximum score of 12 indicates the worst. The macroscopic appearance of the wound was recorded daily with a camera (Canon, Tokyo, Japan).

### 4.8. Western Blotting Analysis

The skin tissues were lysed in 1 × SDS sample buffer [50 mM Tris-HCl (pH 7.4), 150 mM NaCl, 0.1% SDS, 2 mM β-mercaptoethanol, 1 mM DTT, BPB, and xylene cyanol]. The tissue lysates were electrophoresed on a 10 or 12% SDS polyacrylamide gel and proteins were transferred to polyvinylidene difluoride (PVDF) membranes at 300 mA for 1 h. After a brief rinse with PBST (PBS containing 0.1% Tween 20), the blots were blocked for 1 h at room temperature in blocking buffer (PBST containing 4% non-fat dried milk). Primary antibodies against anti-collagen type-1 or anti-filaggrin diluted in blocking buffer (1:1,000) were added to the blots and the blots were incubated for 1 h at room temperature or overnight in a refrigerator. After washing three times with PBST, the blots were incubated with HRP-conjugated secondary antibodies (1:10,000 dilutions in blocking buffer) for 1 h at room temperature. The blots were washed three times in PBST and developed with super signal enhanced chemiluminescence (ECL) substrate solution (Pierce, Rockford, IL, USA) for an appropriate time according to the manufacturer’s instructions. The signals were then visualized using X-ray film.

### 4.9. Immunohistochemical Analysis

The skin tissues were fixed with 4% paraformaldehyde, embedded in paraffin and then 5-µm thin sections were made on silane coated micro slides (Muto-glass, Tokyo, Japan). The de-paraffinized skin sections were stained with anti-collagen type-1 and anti-flaggrin. The sections for immunohistochemistry were de-paraffinized wth xylene, rinsed with distilled water and then rinsed with PBS. The nonspecific antigen–antibody reactions were inhibited by one hour treatment with 10% normal goat serum. The sections were reacted overnight at 4 °C in diluted solutions of each of the primary antibodies such as purified rat monoclonal antibodies against anti-collagen typee-1 and anti-filaggrin antibodies. The sections were rinsed with distilled water and PBS and then were covered with cover-slip. They were examined by fluorescence microscopy (Olympus, Tokyo, Japan) to assess the molecular expressions and cell infiltrations.

### 4.10. Statistical Analysis

Differences in data among the groups were analyzed by one-way ANOVA, and all values were expressed as mean ± S.E. The differences between groups were considered to be significant at *p* < 0.05.

## 5. Conclusions

In summary, this study demonstrates that DBC notably accelerates the spreading rate and type 1 collagen production of HaCaT keratinocytes and topical application of DBCNFM significantly increases the skin remodeling in hairless mice through induction of type 1 collagen and filaggrin expression. These results indicate that the DBCNFM can be used as a dressing material for wound healing.
